# Transcriptional analysis of murine biliary atresia identifies macrophage heterogeneity and subset-specific macrophage functions

**DOI:** 10.3389/fimmu.2025.1506195

**Published:** 2025-01-30

**Authors:** Kyle D. Gromer, Shang-Yang Chen, Gaurav Gadhvi, Liang Feng, Colin Shearn, Swati Antala, Joshua B. Wechsler, Carla M. Cuda, Cara L. Mack, Ronald J. Sokol, William J. Janssen, Richard M. Green, Harris Perlman, Deborah R. Winter, Sarah A. Taylor

**Affiliations:** ^1^ Department of Pediatrics, Ann & Robert H. Lurie Children’s Hospital of Chicago, Chicago, IL, United States; ^2^ Department of Medicine, Northwestern University, Chicago, IL, United States; ^3^ Department of Pediatrics, Children’s Hospital Colorado and University of Colorado School of Medicine, Aurora, CO, United States; ^4^ Division of Hepatology, Department of Pediatrics, Kravis Children’s Hospital at Mount Sinai, New York, NY, United States; ^5^ Department of Pediatrics, Children’s Wisconsin, Milwaukee, WI, United States; ^6^ Department of Medicine, National Jewish Health, Denver, CO, United States

**Keywords:** cholestasis, obstructive cholangiopathy, pediatric liver disease, innate immunity, hepatic macrophages

## Abstract

**Introduction:**

Macrophages play an important role in disease progression of pediatric cholestatic liver disease, particularly biliary atresia (BA); however, the restorative versus pathogenic role for precise macrophage subsets remains poorly defined. We aimed to distinguish the transcriptional profiles and roles of defined macrophage subset(s) in murine BA.

**Methods:**

We used multiparameter flow cytometry and RNA-sequencing analysis to profile recruited CD11b^hi^CD64+ hepatic macrophages by cell surface expression of MHCII and Ly6c in the Rhesus rotavirus (RRV)-induced murine model of BA versus saline controls. Modulation of macrophage numbers via intra-peritoneal injections of clodronate-loaded liposomes was performed to determine the association between macrophage numbers and histologic injury (Ishak score).

**Results:**

Ly6c+ macrophages demonstrated the greatest increase in numbers and percent of total macrophages in murine BA versus saline controls whereas MHCII+ macrophages decreased. Transcriptional changes in murine BA MHCII+ macrophages included reduced expression of the Kupffer cell gene signature, lower expression of genes involved in homeostatic processes, and increased expression of genes involved in inflammatory processes. Ly6c+ macrophages in murine BA showed increased expression for *Hif1a* and other genes involved in the cellular response to hypoxia. Among all subsets, the number of Ly6c+ macrophages exhibited the strongest correlation with severity of histologic liver injury by Ishak score.

**Conclusions:**

Our data identify specific pathways upregulated in Ly6c vs MHCII+ macrophage subsets in murine BA. Transcriptional similarities between murine BA and human cholestatic macrophages may enable translation of future mechanistic studies to new macrophage subset-specific therapies.

## 1 Introduction

Biliary atresia (BA) is a cholestatic liver disease of infancy that remains the leading cause of pediatric liver transplantation due to a lack of effective medical therapies. Part of the difficulty in establishing new medical therapies is the multifactorial disease pathogenesis including a complex immune response (1). Current evidence supports the principle that BA arises from an aberrant immune response to an environmental trigger (1). However, the exact immune mechanism of disease onset and progression remains unknown.

Macrophages are critical cells within the innate immune system that have been shown to play a key role in disease pathogenesis of BA. Increased number of macrophages in portal tracts on histology has been associated with poor prognosis in infants with BA after Kasai portoenterostomy (2–5). Similarly, macrophage numbers increase in the Rhesus group A rotavirus (RRV)-induced murine model of BA (6), and RRV has been shown to target hepatic macrophages (7). Additionally, macrophage depletion using clodronate in a mouse model of cystic BA (Stat1^-/-^) ameliorated bile duct obstruction (8), while disruption of IL-17A signaling reduced hepatic macrophage recruitment and improved survival in murine BA (9). On the other hand, macrophages play a critical role in maintaining host tolerance to non-pathogenic gut-derived antigens in homeostasis (10). Recently our understanding of hepatic macrophage heterogeneity has expanded with the advent of single-cell technology (11–15), however, prior studies in BA have not fully leveraged this technology to define the distinct pathogenic macrophage subset(s).

Macrophages are heterogeneous and highly plastic cells that can adopt diverse functional phenotypes in response to specific environmental cues (16). Macrophage polarization can be characterized based on their role in inflammation as well as other functional states such as angiogenesis or wound healing (16). To begin to understand the role for macrophage heterogeneity in pediatric cholestatic liver disease, we previously leveraged single-cell RNA-sequencing (scRNA-seq) technology to define three macrophage populations with distinct transcriptional signatures in children with cholestasis at the time of liver transplant (11). We labeled these subsets as lipid-associated macrophages (LAM; high expression for genes in lipid metabolism similar to previously identified adipose tissue macrophages (17)), monocyte-like macrophages (MLM; increased expression of genes associated with monocytes), and adaptive macrophages (AM; enrichment for genes involved in lymphocyte activation) (11). These subsets were transcriptionally distinct from macrophages found in healthy adults and children (11, 13). However, variability across individuals and limitations in availability of samples, restricted our ability to directly compare healthy and diseased macrophages at similar timepoints.

In the present study, we overcome this gap in knowledge by extending findings from our human work to the well-established RRV-induced murine model of BA. We use a combination of multiparameter flow cytometry and RNA sequencing (RNA-seq) to profile macrophage subsets in murine BA, gain insight into their ontogeny, and compare them to human cholestatic macrophages. We use flow cytometry to differentiate recruited CD11b^hi^ versus tissue resident CD11b^lo^ macrophages. We further characterize CD11b^hi^ macrophage subtypes by cell surface expression of Ly6c given the established pro-inflammatory nature of Ly6c+ macrophages versus the more anti-inflammatory Ly6c- macrophages involved in functions such as wound healing (18). We also characterize macrophages by MHCII cell surface expression to identify the extent to which macrophages may play a role in antigen-presentation in BA, as has been described (1). We demonstrate a loss of tissue resident macrophages in murine BA, reduced expression of the tissue resident transcriptional signature in recruited murine BA macrophages, and increased number of Ly6c+ macrophages that are directly associated with the severity of liver injury. By characterizing the pathogenic macrophage subsets in murine BA, our study will enable future translational studies to investigate similar human pathways and explore new therapeutic targets to improve patient outcomes.

## 2 Materials and methods

### 2.1 RRV-induced murine model of BA

BALB/c mice were purchased from Jackson Laboratory (Catalog #000651) and housed and bred within Northwestern University’s Center for Comparative Medicine. All mouse experiments were approved by the Northwestern University Institutional Animal Care and Use Committee. Newborn mice received an intra-peritoneal injection within 24 hours of life of either 50 μl RRV (1.8x10^6 pfu) to establish murine BA or 50 μl phosphate buffered saline (PBS) for controls (Gibco Catalog #10010023) (19). Blood and histology samples were obtained from both experimental groups at DOL 14 to confirm sequelae of biliary obstruction and quantify F4/80-positive macrophages. Paraffin-embedded liver tissue was stained for F4/80 (Cell Signaling #70076 at 1:750) in the Northwestern University Mouse Histology and Phenotyping Laboratory. Slides were assessed on a Nikon 80i (Nikon, Melville, NY, USA) microscope with a DS-Ri2 color camera with Nikon NIS-Elements Advanced Research software (version 5.21.02). At least three 20x images of portal tracts were captured for each mouse, and the number of cells per mm^2^ were determined in a blinded fashion using the same algorithm for all samples (investigators JW and SA). Statistical comparison of cell numbers on histology between the experimental model and control were made by unpaired t-test in GraphPad Prism version 9.1.2. Further evaluation of macrophage numbers by flow cytometry was accomplished using liver tissue from female mice of each group at DOL 7, DOL 10, and DOL 14; transcriptional analyses were performed at DOL 14 (described below); outcome after clodronate administration was evaluated at DOL 12 (described below). Female mice were chosen to limit differences from sex on transcriptional analyses and used for consistency throughout experiments.

### 2.2 Liver tissue digestion and flow cytometry

Each liver sample was cut into small pieces in a c-tube (Miltenyi Catalog #130-093-237) and 2.5 mL of digestion buffer was added: 2 mg of DNase I (Sigma #10104159001), 585 mL of Liberase TL (Sigma #5401020001), and 9.215 mL of HBSS (Gibco #14025076). Additional tissue digestion was achieved using both the Miltenyi Biotec gentleMACS Dissociator (non-clodronate experiments only) and incubation with shaking at 37°C for 30 minutes. We next strained the liver homogenate through a 40 μm filter, rinsing with HBSS to a total volume of 30 mL and spun the samples at 400 rcf for 7 minutes (4°C). The pellet was resuspended in Pharm Lyse (Becton Dickinson #555899) to lyse remaining red blood cells. Two additional washes using HBSS and a 40 μm filter were performed prior to enrichment with CD45 MicroBeads and magnetic separation with Multi-24 Columns (Miltenyi, 130-095-691) on the MACS Separator following the manufacturer’s guidelines (Miltenyi, Bergisch Gladbach, Germany #130-052-301). Cell count was performed upon completion of enrichment to determine the appropriate amount of live/dead stain (Invitrogen, 0.3 μl live/dead stain plus 500 μl HBSS per 5.0x10^6 cells), FC Block (Becton Dickinson, 6 μl of FC Block plus 54 μl of MACS Buffer per 5.0x10^6 cells) and fluorescent antibodies (**Supplementary Table 1**, 40 μl total volume per 5.0x10^6 cells). Cells were washed and filtered with MACS buffer and a 40 μm filter after which multiparameter flow cytometry identified immune cell subsets of interest (**Supplementary Figure 1**).

The BD FACSAria at the Robert H. Lurie Comprehensive Cancer Center Flow Cytometry Core Facility was utilized for flow cytometry and sorting. Our gating strategy was established using our previously published flow gating strategy on CD11b^hi^ macrophages that was developed with fluorescence minus one controls (20), prior single-cell sequencing data on CD11b^hi^ and CD11b^lo^ macrophages (20), and unpublished data from antibody-derived tags (ADT) for CD11b (BioLegend TotalSeq™-A, clone M1/70), F4/80 (BioLegend TotalSeq™-A, clone BM8), and CD64 (BioLegend TotalSeq™-A, clone X54-5/7.1) (**Supplementary Figure 1**). These data demonstrated that 2 of 3 macrophage subsets in healthy mice at DOL 14 have low expression of *Itgam* (CD11b) but high *Adgre1* (F4/80), *Clec4f*, and *Timd4* expression in line with tissue resident macrophages, i.e. Kupffer cells (KCs). We therefore used our flow cytometry panel to label Cd11b^lo^F4/80+ cells as KCs. In contrast to healthy macrophages, all murine BA macrophage subsets showed high *Itgam* (Cd11b) expression supporting a loss of KCs. To further define the role for recruited macrophages in BA and determine if these recruited subsets may transition to a KC phenotype, we sorted CD11b^hi^ macrophages for downstream RNA sequencing analysis and compared these subsets to their non-diseased counterparts.

Fluorescence-activated cell sorting (FACS) isolated CD11b+CD64+ macrophages by expression of Ly6c and MHCII as previously established (20). This led to 4 populations: Ly6c+MHCII+, Ly6c+MHCII-, Ly6c-MHCII+, and Ly6c-MHCII- macrophages which were annotated double positive (DP), Ly6c+, MHCII+, and double negative (DN) respectively. A range of 99,304 to 223,262 and 236,871 to 523,715 total macrophages were sorted for each replicate of saline and murine BA mice respectively. FlowJo 10.6.2 software was used for analysis of all flow data. Cell numbers were determined using count beads (123count eBeads Counting Beads, Invitrogen, Catalog # 01-1234-42). Comparisons of cell numbers between experimental groups across time-points (or between clodronate-treated vs. saline) was performed by ANOVA with Bonferroni correction and one-tailed unpaired Welch’s t-test as appropriate with significance defined as p-value < 0.05 (GraphPad Prism).

### 2.3 Bulk RNA sequencing analysis

RNA Libraries for all RRV and saline samples were extracted, synthesized, and sequenced in one batch. Extractions were performed using the PicoPure RNA Isolation Kit (Catalog #KIT0204). Full-length cDNA libraries were synthesized using the NEBNext Ultra RNA Library Prep Kit (New England BioLabs) followed by TruSeq DNA HT Sample Prep Kit (Illumina) and sequenced by Illumina NextSeq500 to an average depth of ~15x10^6^ reads per sample. The resulting BCL files were demultiplexed into fastq files which were then trimmed for low quality bases and adaptors using Trimmomatic (https://github.com/usadellab/Trimmomatic). Trimmed reads were aligned to the mm10 reference using Tophat (21, 22) and mapped to gene exons in the Mus_musculus.GRCm38 reference with HTseq (23). Gene expression counts were normalized to read depth using counts per million reads mapped (CPM). To filter out lowly expressed genes, genes with log 3.5 CPM in at least 2 samples were excluded from the analysis resulting in 10,605 expressed genes. Replicates were excluded from analysis based on low read depth or concern for contamination (1 each of saline and RRV DN samples). Principal Component Analysis (PCA) and Pearson’s correlations were computed on expressed genes and visualized using ggplot2. To define highly variable genes across saline-treated macrophage subsets, we performed ANOVA-based filtering with adjusted P-value < 0.01 (Benjamini-Hochberg) and identified 3,200 genes. K-means clustering (k=6) was performed in Morpheus (https://software.broadinstitute.org/morpheus/) using the highly variable genes across saline samples and visualized for both saline and RRV samples by normalizing into Z-scores. DESeq2 package (24) was utilized for the identification of pairwise differentially expressed genes (DEGs) between saline and RRV conditions for each macrophage subset. DEGs were defined as filtered genes expressed in the relevant subset with adjusted P-values < 0.05 and |log2FC| > 1. Volcano plots were generated using the EnhancedVolcano package, and the overlap between DEGs from each comparison were computed and plotted via the UpsetR package. Gene ontology (GO) enrichment analysis was executed using GOrilla (25) on each saline gene cluster with all highly variable genes as background (3200 genes) as well as independently on up- and down-regulated DEGs between murine BA and saline controls (total expressed genes used as background). P-value threshold to identify significant GO processes within GOrilla was set to p < 10^-3^. Enrichment for specific processes of interest using the GSVA package (26) included Hallmark Reactive Oxygen Species (27), Hallmark Angiogenesis (27), Mouse Genome Database Phagocytosis engulfment (28, 29), and Senescence (30).

DEGs during the differentiation from circulating monocytes into Kupffer cells (KC) were retrieved from Sakai et al., 2019 (31) and defined as those with adjusted p-values < 0.05 and |log2FC| > 1. Hypergeometric enrichment was calculated comparing the overlap of these DEGs with 6 K-means clusters using custom dataset function in MAGNET (32). Genes that characterized macrophages in nonalcoholic steatohepatitis (NASH, now referred to as metabolic dysfunction-associated steatohepatitis or MASH) were identified based on differentially expressed gene sets defined by Seidman et al (33). The overlap between recruited macrophage subsets in MASH and DEGs increased in murine BA macrophage subsets was determined using the online tool ‘Venny’ (34).

We next evaluated the transcriptional similarities between macrophage subsets in RRV-induced murine BA and previously published data from human and murine cholestatic macrophages. For comparison to human macrophage subpopulations from previously published scRNA-seq data, the top 20 markers from adult control macrophages (13) and pediatric cholestatic macrophages were converted to their murine orthologs (11) and their mean expression in macrophage subsets was Z-score normalized. Further comparison was performed between our bulk RNA-seq data and the top differentially expressed genes with average Log2FC > 1 and p-value > 0.05 as identified by prior single-cell sequencing analysis of murine cholestatic macrophages (murine BA and neonatal bile duct ligation) and non-diseased neonatal mice (20). Enrichment was assessed and visualized by gene set variation analysis using the GSVA package (26).

### 2.4 Clodronate administration in murine BA

Clodronate-loaded liposomes were used to assess macrophage depletion in murine BA and the effect on disease phenotype. Intra-peritoneal injections of clodronate-loaded liposomes (100 μl, Liposoma, #C-005) versus PBS (100 μl, Gibco #10010023) were performed in healthy BALB/c pups at DOL 3, 6, and 9 to establish the efficacy of macrophage depletion. Next, the same protocol of clodronate-loaded liposomes versus PBS was administered to the murine model of BA. Changes in liver histology, blood chemistry, and hepatic immune cell subsets (flow cytometry) were evaluated at DOL 12. Ishak scoring was performed on hematoxylin and eosin stained slides from all mice by two blinded investigators (CS and ST) as previously described (35). Ishak scores are comprised of categories for piecemeal necrosis characterized by periportal or periseptal interface hepatitis (score 0-4), confluent necrosis (score 0-6), focal lytic necrosis (score 0-4), and portal inflammation (score 0-4) (36). The average score per category and total Ishak score were compared between experimental groups. Pearson correlation was used to determine if there was an association between histologic injury and macrophage number.

## 3 Results

### 3.1 Increased number of Ly6c+ macrophages are present in BA

**Figure 1:**
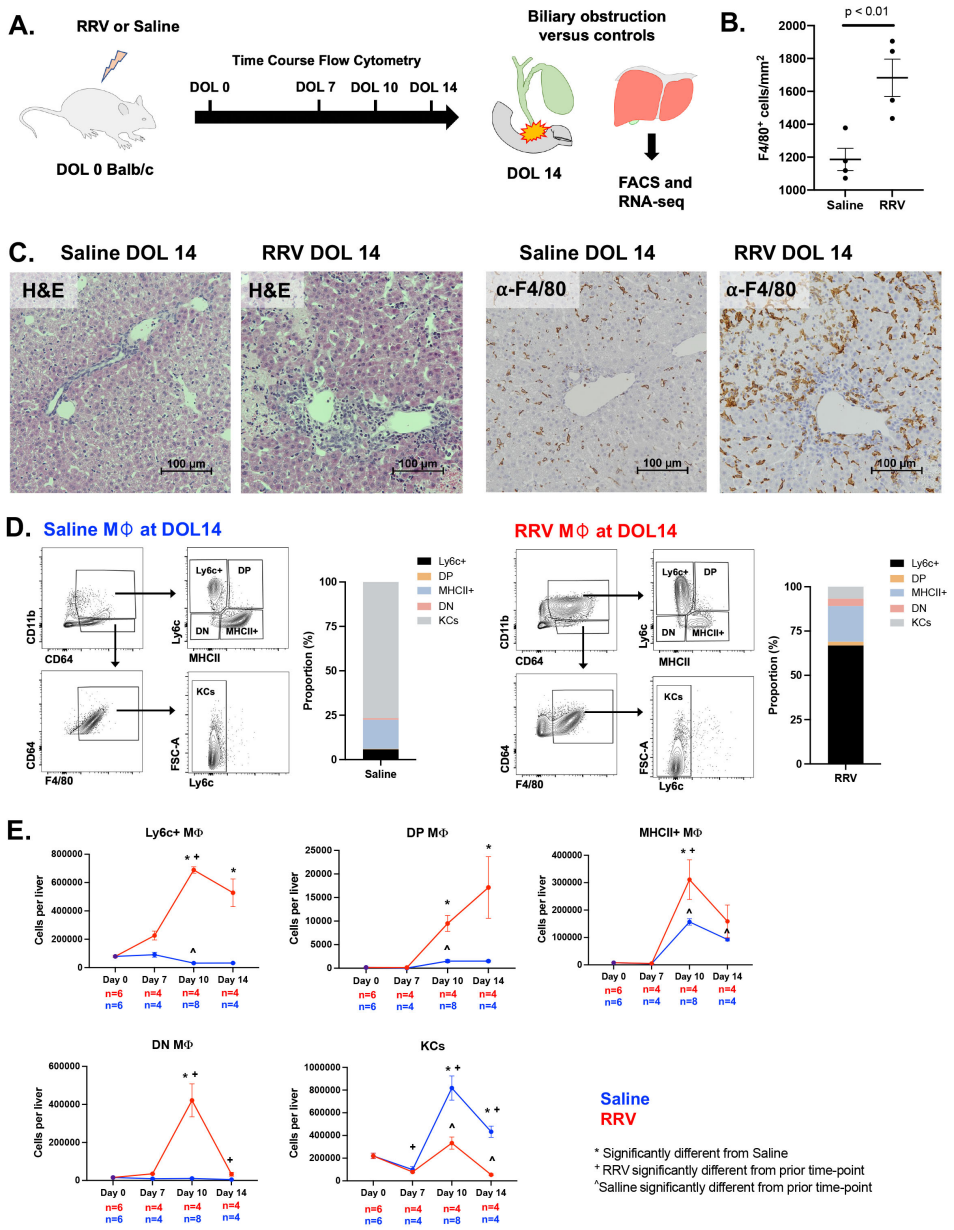
Compositional changes in murine BA macrophage subsets. **(A)** Overview of experimental design for flow cytometry and RNA-sequencing analyses in murine BA mice and saline controls. **(B, C)** Increased F4/80+ macrophage infiltration is present in murine BA with associated hepatic necrosis. Hematoxylin and eosin staining shows inflammation and liver injury characterized by hepatic necrosis. **(D, E)** Flow cytometry analysis over the first 14 days of life shows changes in numbers of macrophage subsets in murine BA versus saline controls as defined by cell surface expression of MHCII and Ly6c. The average number of macrophages across replicates was calculated and the proportion of macrophages compared between experimental groups. At day of life 14, KCs and CD11b^hi^ MHCII+ macrophages comprised the majority of total macrophages in saline controls as compared to CD11b^hi^ Ly6c+ macrophages in murine BA **(D)**. ANOVA with Bonferroni correction was performed and p-values < 0.05 for comparisons between experimental groups and prior time points within experimental groups are shown **(E)**. DOL, day of life; DN, double negative; DP, double positive; RRV, Rhesus rotavirus.

Following the experimental design depicted in **Figure 1A**, we confirmed hepatocellular injury, portal inflammation, and bile duct injury in RRV-induced murine BA by hematoxylin and eosin stain of liver histology at day of life (DOL) 14 (**Figure 1C**). Staining and quantification of total F4/80+ macrophages showed increased numbers in murine BA (**Figures 1B, C**). We used the flow cytometry panel outlined in **Supplementary Figure 1** to define four subpopulations of CD11b^hi^CD64+ macrophages by the cell surface expression of Ly6c and MHCII as well as CD11b^lo^F4/80+ KCs. Evaluation of these subsets by flow cytometry at DOL 14 showed that KCs comprised 77% of total macrophages in controls followed by MHCII+ macrophages at 16%. In contrast, Ly6c+ macrophages comprised the majority of total macrophages (67%) in murine BA whereas KCs comprised only 7% (**Figures 1D, E**). More specifically, Ly6c+ macrophages in murine BA increased in number from DOL 0 to DOL 14 by 6.7 times whereas they decreased in number in saline controls. DP and DN also increased in number from DOL 0 to DOL 14 in murine BA (**Figure 1D, E**). Importantly, KCs were present in similar numbers between experimental groups at DOL 7 suggesting depletion in murine BA rather than delayed development of KCs in the RRV model. In addition to KC loss, these data show that disease progression is accompanied by compositional changes in the CD11b^hi^ macrophage pool in murine BA.

### 3.2 Macrophage transcriptional heterogeneity is reduced in murine BA

**Figure 2:**
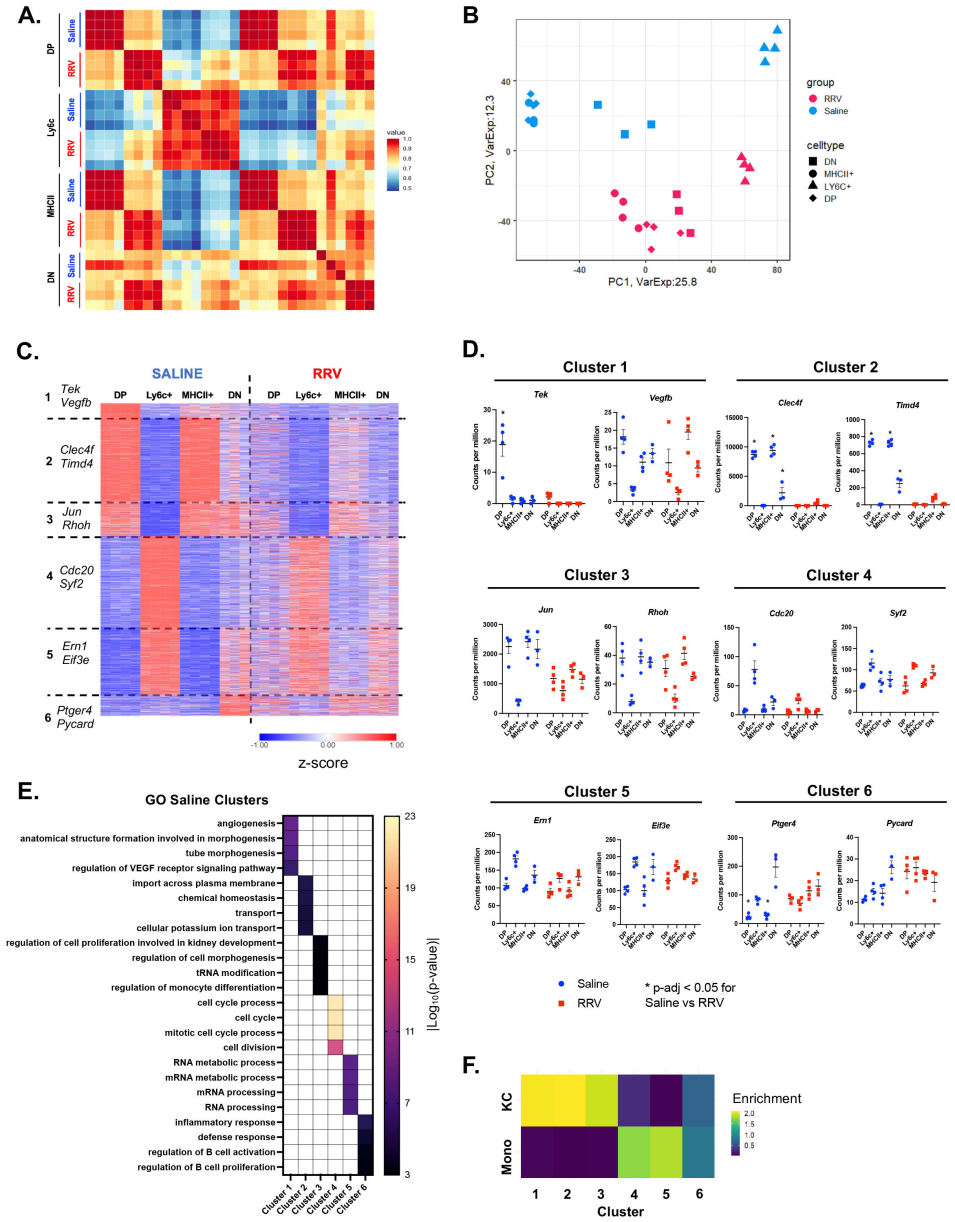
Reduced transcriptional heterogeneity is present across murine BA macrophages. **(A)** Pearson correlation of expressed genes demonstrates similar transcriptional profiles between corresponding macrophage subsets in saline controls and murine BA. **(B)** Visualization of expressed genes by principal component analysis shows reduced heterogeneity among murine BA macrophages. **(C)** K-means clustering of 3,200 highly variable genes identified 6 gene clusters that differentiated saline macrophage subsets but were expressed at lower levels in murine BA macrophages. **(D)** Representative genes that differentiate each saline cluster are shown for both experimental models. **(E)** Gene ontology enrichment analysis for genes of each saline cluster identified distinct processes for each cluster. **(F)** Comparison of all saline gene clusters to previously defined differentially expressed genes (DEGs) of circulating monocytes and KCs showed greatest enrichment for KC genes in clusters 1-3 as compared to enrichment for monocyte genes in clusters 4-5 (31). n=4 for all transcriptional comparisons except DN subsets where n=3. * indicates adjusted p-value < 0.05 by DESeq. DN, double negative; DP, double positive; KC, Kupffer cell; Mono, monocyte; RRV, Rhesus rotavirus; VEGF, vascular endothelial growth factor.

We next evaluated the transcriptional variability across CD11b^hi^ macrophage subsets to understand their functional significance in disease pathogenesis. We observed that the macrophage subsets of saline mice demonstrated greater heterogeneity as compared to murine BA (**Figures 2A, B**). K-means clustering of the highly variable genes across the 4 saline macrophage subsets identified 6 gene clusters associated with distinct pathways (**Figures 2C–E**; **Supplementary Tables 2**, **3**). Cluster 1, which was most highly expressed in DP macrophages, was enriched for genes involved in angiogenesis and development (e.g. *Tek*, *Vegfb*) whereas cluster 2, which was shared between the DP and MHCII+ subsets, was associated with transport, chemical homeostasis, and catabolic processes (e.g. *Clec4f*, *Timd4*). Cluster 3 was expressed in all subsets except Ly6c+ macrophages and contained genes such as *Jun* and *Rhoh* involved in the regulation of monocyte differentiation and cell morphogenesis. On the other hand, Ly6c+ macrophages had the greatest expression of genes in clusters 4 and 5 that were associated with cell cycle (e.g. *Cdc20*, *Syf2*) and regulation of gene expression and RNA metabolic processes (e.g. *Ern1*, *Eif3e* respectively). Lastly, a small set of genes (cluster 6) differentiated saline DN macrophages from the other 3 macrophage subsets and were enriched in defense response and regulation of cell activation (e.g. *Ptger4*, *Pycard*).

To gain insight into how these pediatric macrophages relate to known adult subpopulations, we compared our clusters to previously published data on the transition of circulating monocytes to tissue-resident KCs (31). The greatest enrichment for the KC gene signature was observed in saline clusters 1-3 that was associated with MHCII+ and DP macrophages. In contrast, the monocyte gene set was enriched in saline clusters 4 and 5 that differentiated Ly6c+ macrophages (**Figure 2F**). DN macrophages (defined by cluster 6) were not associated with either circulating monocytes or KC gene signatures suggesting a possible transitioning subset. In agreement with these results, we found that *Clec4f* and *Timd4*, genes expressed by tissue resident KCs derived from embryonic progenitors (37, 38), were highest in Saline Cluster 2 that defined saline DP and MHCII+ macrophages (**Figures 2C, D**). The overall patterning of gene expression across macrophages in murine BA was similar to controls although the differences between subsets were reduced (**Figure 2C**). This was particularly evident by decreased expression of clusters 1-3 in DP and MHCII+ (**Figures 2C, D**). These data suggest that saline CD11b^hi^ DP and MHCII+ macrophages may be adopting a canonical tissue-resident macrophage signature whereas Ly6c+ macrophages are more similar to monocyte-derived macrophages. In contrast, murine BA hepatic macrophages do not adopt a tissue-resident signature leading to reduced heterogeneity across subpopulations.

### 3.3 Murine BA macrophages exhibit unique transcriptional signatures

**Figure 3:**
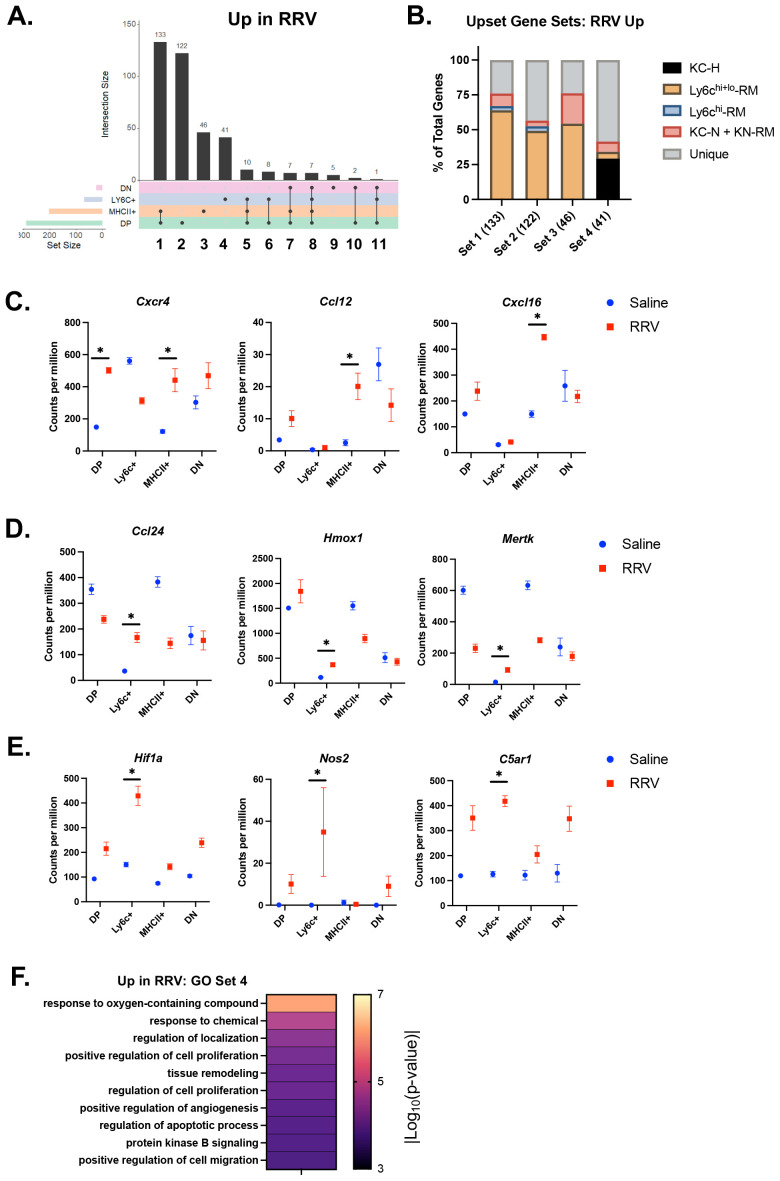
Inflammatory processes are upregulated in murine BA macrophages. **(A)** The number of differentially expressed genes (DEGs) with increased expression in murine BA versus saline controls and the overlap between subsets (solid line) was calculated using the UpsetR package. Overall, MHCII+ and DP macrophage subsets showed the greatest number of increased DEGs. **(B)** Evaluation of the overlap between DEGs in gene sets 1-4 and previously identified genes in macrophages from a murine model of MASH (33) demonstrated the highest proportion of unique genes present in gene set 4 that defined murine BA Ly6c+ macrophages. **(C)** Immune signaling and pro-inflammatory genes increased in murine BA MHCII+ and DP macrophages included *Cxcr4*, *Ccl12*, and *Cxcl16*. **(D)** Genes present in set 4 that defined murine BA Ly6c+ macrophages that were also present in healthy KCs included *Ccl24*, *Hmox1*, and *Mertk.* **(E)** Set 4 genes not present in any MASH subsets included *Hif1a*, *Nos2*, *C5ar1*. **(F)** Evaluation of enriched processes from gene set 4 identified processes related to hypoxia and angiogenesis. n=4 for all transcriptional comparisons except DN subsets where n=3. * indicates adjusted p-value < 0.05 by DESeq. DN, double negative; DP, double positive; KC-H, healthy KC; KC-N, NASH KC; KN-RM, recruited macrophage occupying the KC niche; RM, recruited macrophage; RRV, Rhesus rotavirus.

As hepatic macrophages can adopt novel functions based on disease-specific environmental cues (16), we evaluated DEGs in murine BA versus saline controls for each macrophage subset and identified overlapping gene sets (**Supplementary Figure 2**, **Supplementary Tables 4**, **5**). As seen in **Figure 2**, saline gene clusters associated with the KC signature and enriched for homeostatic processes were decreased in expression in MHCII+ and DP murine BA subsets (**Supplementary Figure 3A**, **Supplementary Table 6**). Among increased DEGs in murine BA, we found that the top three gene sets were either shared or unique to MHCII+ and DP macrophages (**Figure 3A**). Comparison to genes that characterized macrophage subpopulations in another mouse model of disease (murine MASH) showed > 50% overlap for each of the top 3 gene sets with recruited macrophages (RM) in MASH suggesting upregulation of these genes was not disease-specific (**Figure 3B**). Overall, these top three gene sets were enriched in inflammatory processes such as regulation of cell adhesion and response to interferon-gamma (*Cxcr4, Ccl12*, *Cxcl16*) (**Figure 3C**; **Supplementary Figure 3B**; **Supplementary Table 7**).

The 4^th^ gene set comprising 41 DEGs exhibited increased expression only in Ly6c+ macrophages (**Figure 3A**; **Supplementary Table 5**). Within this gene set, 59% of genes were not shared with macrophage subpopulations in murine MASH vs control experiment (**Figure 3B**). Of the ones that were shared, they were primarily found in healthy KCs rather than MASH macrophages (**Figure 3B**). Genes common to Ly6c+ macrophages and healthy KCs included *Ccl24*, *Hmox1*, and *Mertk* (**Figure 3D**). Unique genes within this gene set included genes involved in response to oxygen-containing compound and angiogenesis (*Hif1a*, *Nos2*, *C5ar1*) (**Figure 3E**; **Supplementary Table 7**). Further evaluation of enrichment for gene sets associated with the macrophage response to oxidative injury showed greatest enrichment for the reactive oxygen species pathway as compared to angiogenesis, senescence, and phagocytosis (**Figure 3F**; **Supplementary Figure 3C**) (27–30). These findings support a role for Ly6c+ macrophages in the processes related to tissue hypoxia that may be distinct from macrophage function in non-cholestatic etiologies of liver injury (e.g. MASH).

Lastly, DN macrophages had the smallest number of unique DEGs that were either increased or decreased in murine BA. Furthermore, DN macrophages showed reduced expression of the saline gene cluster 6 characterized by processes involved in defense response. This finding suggests that DN macrophages may lose a potential regulatory function and/or they may be a transitioning subset during the disease course of murine BA. Further research with RNA-sequencing analysis at peak DN macrophages numbers (DOL 10) will help delineate their role.

We next compared expression levels of top DEGs from our prior single-cell sequencing analysis of murine cholestatic macrophages (murine BA and neonatal bile duct ligation) and non-diseased neonatal mice to our Ly6c vs MHCII+ subsets (**Supplementary Figure 4**) (20). This analysis demonstrated the greatest similarity between saline DP and MHCII+ macrophages and normal single-cell clusters 1 and 3 whereas saline Ly6c+ macrophages were similar to normal cluster 6 and cholestatic cluster 3 (**Supplementary Figure 4A**). We show the expression level of representative genes that defined saline DP and MHCII+ macrophages (*Clec4f*, *Timd4*), and saline Ly6c+ macrophages (*Syf2*, *Eif3e*) across the single-cell subsets in **Supplementary Figure 4C**. Among our murine BA macrophages, we found that Ly6c+ macrophages were similar to cholestatic single-cell cluster 0 whereas MHCII+ macrophages were similar to cholestatic clusters 1 and 2 and normal clusters 1 and 3 (**Supplementary Figure 4B**). Expression level of genes increased in murine BA MHCII+ macrophages (*Cxcr4*, *Cxcl16*) and Ly6c+ macrophages (*Hif1a*, *C5ar1*) is shown across all single-cell clusters in **Supplementary Figure 4D**. Overall, this analysis demonstrates transcriptional similarity between our previously published single-cell clusters and macrophage subsets defined by MHCII and Ly6c surface expression in the current study.

### 3.4 Murine BA hepatic macrophages are transcriptionally similar to specific human pediatric cholestatic macrophage subsets

**Figure 4:**
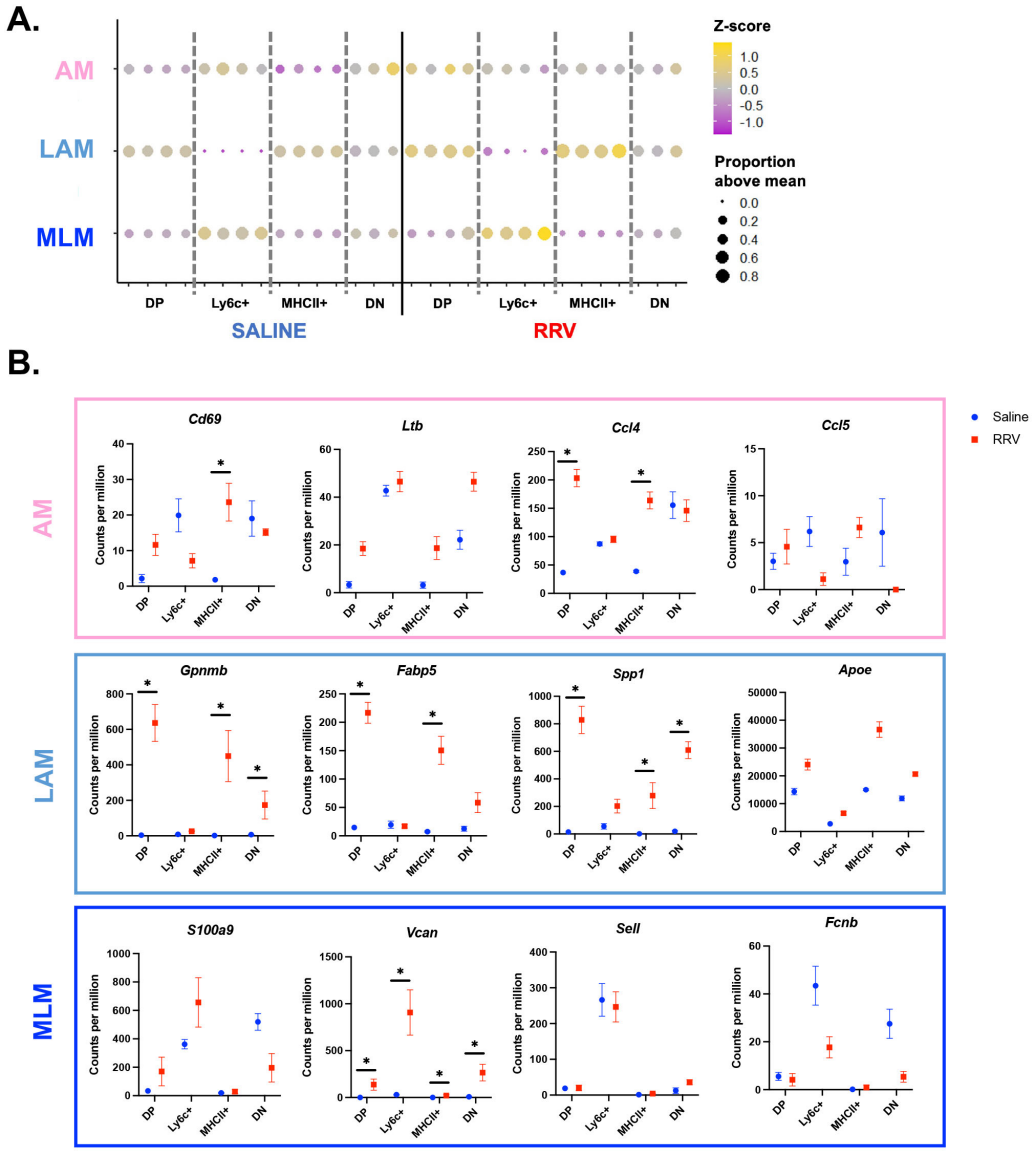
Murine BA macrophages are transcriptionally similar to their human counterparts. **(A)** The top 20 genes from pediatric cholestatic macrophage subsets (11) were converted to their murine orthologs. Evaluation of the mean expression of these genes across murine BA macrophage subsets showed a high level of similarity between MLM and murine BA Ly6c+ macrophages, and LAM and murine BA DP and MHCII+ macrophages. **(B)** Characteristic genes for each previously identified pediatric cholestatic macrophage subset are visualized for our mouse models. n=4 for all transcriptional comparisons in murine experiments except DN subsets where n=3. * indicates adjusted p-value < 0.05 by DESeq. AM, adaptive macrophage; DN, double negative; DP, double positive; LAM, lipid associated macrophage; MLM, monocyte-like macrophage; RRV, Rhesus rotavirus.

We next determined whether neonatal murine BA macrophage subsets align with those described in pediatric cholestatic liver disease patients (11) (**Figure 4**). We found that murine BA DP and MHCII+ macrophages were most similar to LAM with increased expression of *Gpnmb*, *Fabp5*, *Spp1*, and *Apoe* in the disease model (**Figures 4A, B**). Ly6c+ murine macrophages exhibited highest expression of cholestatic MLM genes including *S100a9* and *Vcan* (**Figures 4A, B**). Finally, cholestatic AM genes were not specifically associated with any murine population although they tended to increase in murine BA macrophages compared to saline controls (**Figure 4B**). We also compared the transcriptional profile of our murine macrophage subsets to previously identified adult non-inflammatory (A-NM) and inflammatory (A-IM) macrophages from non-diseased liver (13). As expected, this comparison showed the greatest similarities between A-NM and the MHCII+ and DP saline subsets whereas A-IM were similar to Ly6c+ macrophages across mouse models (**Supplementary Figure 5**). These results suggest that murine BA macrophages parallel macrophage variability observed in pediatric cholestatic liver disease.

### 3.5 Numbers of Ly6c+ macrophages are most significantly correlated with higher severity of liver injury in murine BA

**Figure 5:**
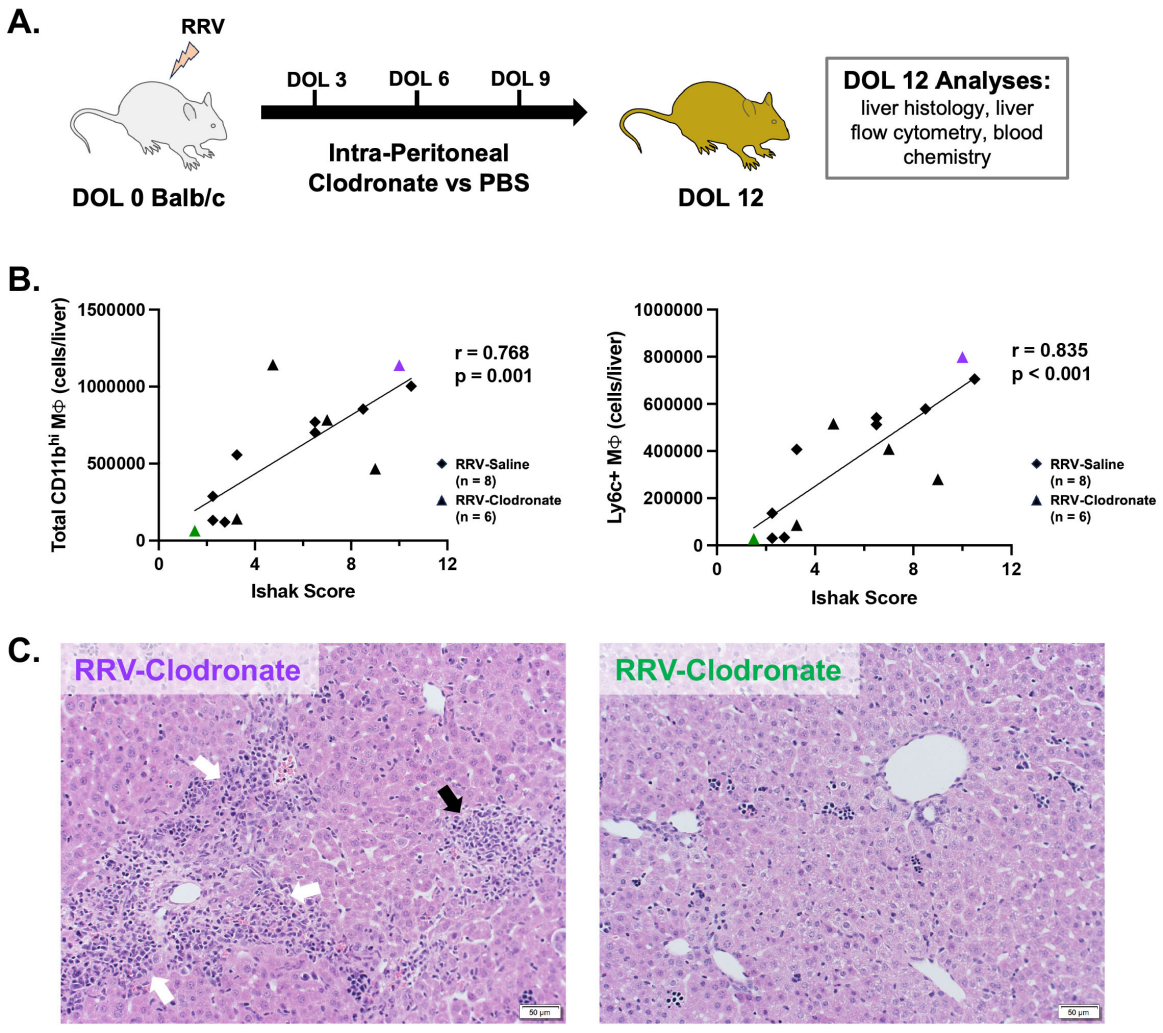
Modulation of macrophage composition in murine BA demonstrates a direct association between Ly6c+ macrophages and severity of histology liver injury. **(A)** Murine BA mice were treated with either 3 intra-peritoneal injections of clodronate-loaded liposomes (n = 6) or PBS (n = 8). Outcome analyses were performed at day of life 12. **(B)** In murine BA mice given intra-peritoneal clodronate versus saline, the total number of CD11b^hi^ macrophages was directly related to the severity of histologic injury by total Ishak score (Pearson’s r = 0.768, p = 0.001). Among all subsets, the number of Ly6c+ macrophages was most strongly associated with liver injury by the Ishak score (Pearson’s r = 0.835, p < 0.001). **(C)** Representative hematoxylin and eosin staining is shown for a clodronate-treated mouse with high macrophage numbers and increased Ishak score (Left, purple triangle in panel **B**) versus low macrophages and Ishak score (Right, green triangle in panel **B**). White arrows show portal inflammation and black arrow shows lobular inflammation. DOL, day of life; PBS, phosphate buffered saline; RRV, Rhesus rotavirus.

To determine how specific CD11b^hi^ macrophage subsets may impact disease severity of murine BA, we studied the effect of clodronate-loaded liposomes on macrophage numbers and disease phenotype (**Figure 5A**). Clodronate-loaded liposomes induce macrophage apoptosis after ingestion of liposomes; clodronate treatment has been shown to deplete circulating monocytes as well as tissue resident macrophages in the adult mouse liver (39–42). In line with these studies, we found that clodronate significantly reduced the number of KCs in both healthy and RRV-treated mice (**Supplementary Figures 6A, B**). Evaluation of CD11b^hi^ macrophage subsets demonstrated a decrease in the proportion of MHCII+ macrophages in healthy DOL 12 mice given clodronate (**Supplementary Figure 6A**). In contrast, clodronate treatment had a variable effect on CD11b^hi^ macrophage subsets in murine BA (**Supplementary Figure 6B**). Clodronate treatment did not significantly reduce neutrophil plus eosinophils in either healthy or BA mice (**Supplementary Figure 6C**). Pooled laboratory data from murine BA mice treated with clodronate versus saline showed no improvement in biochemical markers of obstructive cholestasis with clodronate treatment although statistical analyses could not be performed due to pooling of blood samples (**Supplementary Figure 6D**).

While significant differences in the proportion of individual CD11b^hi^ macrophage subsets were not observed between RRV groups, alterations in the hepatic macrophage niche among clodronate versus saline-treated mice allowed for evaluation of the relationship between the number of macrophages and severity of hepatic injury among murine BA mice. We found that total CD11b^hi^ macrophage number was highly correlated with severity of liver injury by total Ishak score, as well as by portal inflammation, lytic necrosis, and piecemeal necrosis (**Figures 5B, C**; **Supplementary Figure 6E**). Among all subsets, Ly6c+ macrophages had the strongest correlation with total Ishak score (r=0.835; p-value < 0.001) (**Figure 5B**; **Supplementary Figure 6E**). Ly6c+ macrophages were also the only subset significantly associated with each category of hepatic injury (p < 0.05 for all, **Supplementary Figure 6E**). Greater portal and lobular inflammatory injury are shown in the representative image of liver histology (hematoxylin and eosin staining) from a clodronate-treated murine BA mouse with high numbers of Ly6c+ macrophages as compared to reduced portal inflammation in a clodronate-treated mouse with low Ly6c+ macrophage numbers (**Figure 5C**). Taken together, this analysis demonstrates that Ly6c+ macrophages are highly associated with hepatic injury in murine BA.

## 4 Discussion

In the present study, we profile recruited CD11b^hi^ hepatic macrophage subsets in murine BA to better understand the role for early-life macrophage heterogeneity in disease pathogenesis. Among CD11b^hi^ macrophages, we show that the proportion of Ly6c+ macrophages increased the most in murine BA, expressed a *Hif1a*-high gene signature in murine BA, and were strongly associated with the degree of histologic injury. In contrast, the proportion of MHCII+ macrophages decreased in murine BA and lost expression for the tissue-resident macrophage signature supporting impaired capacity to adopt a KC phenotype in disease. These findings extend upon prior data supporting a favorable role for Ly6c Lo macrophages in neonatal RRV-induced injury (43) and supports greater functional heterogeneity of macrophages involved in the disease pathogenesis in BA.

Biliary atresia is characterized by oxidative stress that is thought to contribute to ongoing hypoxic and inflammatory liver injury after disease onset (44–46). Additionally, Hif1-alpha (*Hif1a*), a transcription factor that regulates the hypoxic response, has been shown to be increased in BA cholangiocytes and Hif1-alpha activation can induce the BA phenotype in zebrafish (47, 48). However, prior research has not defined the impact of macrophage-derived *Hif1a* signaling on immune activation in BA. Here, we demonstrate a potential pathogenic role for *Hif1a*-high Ly6c+ macrophages in line with prior research supporting *Hif1a*-driven pro-inflammatory macrophages polarization (49). Our analyses show conflicting results on the degree of enrichment for genes involved in angiogenesis in murine BA Ly6c+ macrophages, a process that can be either a beneficial or maladaptive response to inflammatory hypoxia (50). Importantly, Ccl2-driven recruitment of Ly6c+ macrophages has been shown to play a role in the mechanism of fibrosis-associated angiogenesis in liver disease (51). Further *in vivo* and *in vitro* studies will define the mechanism by which *Hif1a*-high Ly6c+ macrophages respond to oxidative injury in BA and whether they play a role in tissue angiogenesis.

In addition to Ly6c+ macrophages, MHCII+ macrophages also showed a direct association with the severity of histologic liver injury although this association was not as strong. In contrast to saline MHCII+ macrophages, murine BA macrophages had reduced expression for the tissue-resident macrophage signature, supporting a reduced capacity to restore KC function. In parallel with the reduced tissue-resident signature, murine BA MHCII+ macrophages showed reduced expression for genes involved in homeostatic processes and instead demonstrated increased expression for genes involved in cell migration, cytokine production, response to interferon-gamma, and immune system processes. While monocyte-derived macrophages recruited from the bone marrow can repopulate the hepatic macrophage pool after KC loss, this requires complex cell-cell interactions involving hepatocytes, stellate cells, and endothelial cells (31, 37, 52). Chronic inflammation in BA may cause aberrant cell-cell signaling that disrupts macrophage programming toward the tissue-resident transcriptional signature and may prevent restoration of homeostatic function. Future studies defining the specific signaling pathways disrupted in BA may help identify macrophage subset-specific therapeutic targets to slow disease progression and restore the KC macrophage niche.

Central to experimental approaches using murine models of disease is the ability to translate findings to humans. In the present study we showed a high degree of similarity between previously identified human MLM and Ly6c+ murine BA macrophages, suggesting human MLM may play a critical role in disease pathogenesis as we have shown in murine BA (11). Both MHCII+ and DP murine BA macrophages were similar to human LAM that expressed genes involved in lipid metabolism. Additional studies will determine whether dysregulation of MHCII+ and DP macrophages or increased activity of Ly6c+ macrophages are both necessary for tissue injury in BA.

The minimal effect of clodronate-loaded liposomes on subset-specific macrophage depletion in murine BA in our study contrast with prior work in a model of cystic BA using Stat1^-/-^ mice that showed lower macrophage numbers after clodronate administration (8). This observed difference may in part be secondary to the deficient Th1 and interferon-gamma signaling present in Stat1-/- mice. As high interferon-gamma signaling is present in murine BA (53) and interferon-gamma can inhibit macrophage endocytosis (54), it remains possible that reduced endocytic capability of macrophages limited the efficacy of clodronate-loaded liposomes in our study. Alternatively, the large influx of macrophages in murine BA may limit the ability for clodronate-mediated macrophage depletion using standard liposome volumes. Given this limitation our study identifies correlation between macrophage number and severity of liver injury. We also recognize that the RRV-induced murine model of BA more closely replicates inflammatory features rather than the fibrotic changes observed in later stages of human disease. Lastly, our data reflects the transcriptional landscape of Cd11b^hi^ macrophage subsets in murine BA and do not identify transcriptional changes within Cd11b^lo^ KCs in BA. Furthermore, future efforts to optimize our tissue digestion protocol will help reduce the proportion of dead cells that may impact data interpretation.

In conclusion, this study significantly advances our understanding of the impact of macrophage heterogeneity on disease progression in pediatric cholestatic liver disease. We provide evidence to support a role for *Hif1a*-high Ly6c+ macrophages in BA. Our data will lay the foundation for future mechanistic studies to define the precise role for the macrophage-driven response to oxidative stress in disease pathogenesis of BA and alternate etiologies of obstructive cholestasis. Ongoing studies will validate functional similarities between human and mouse subsets and define critical disease-specific cell-cell interactions to enable successful translational initiatives. Ultimately our work may help identify new therapeutic strategies to promote a pro-restorative phenotype of macrophage subsets in BA.

## Data Availability

The data presented in the study are deposited in the Gene Expression Omnibus repository, accession GSE287345.
