# Supplementary material for: Transcriptional analysis of murine biliary atresia identifies macrophage heterogeneity and subset-specific macrophage functions
Source: Front Immunol. 2025 Jan 30;16:1506195. doi: 10.3389/fimmu.2025.1506195 (PMC11821939; doi:10.3389/fimmu.2025.1506195)
Supplement: Supplementary file 8 [file DataSheet1.pdf]

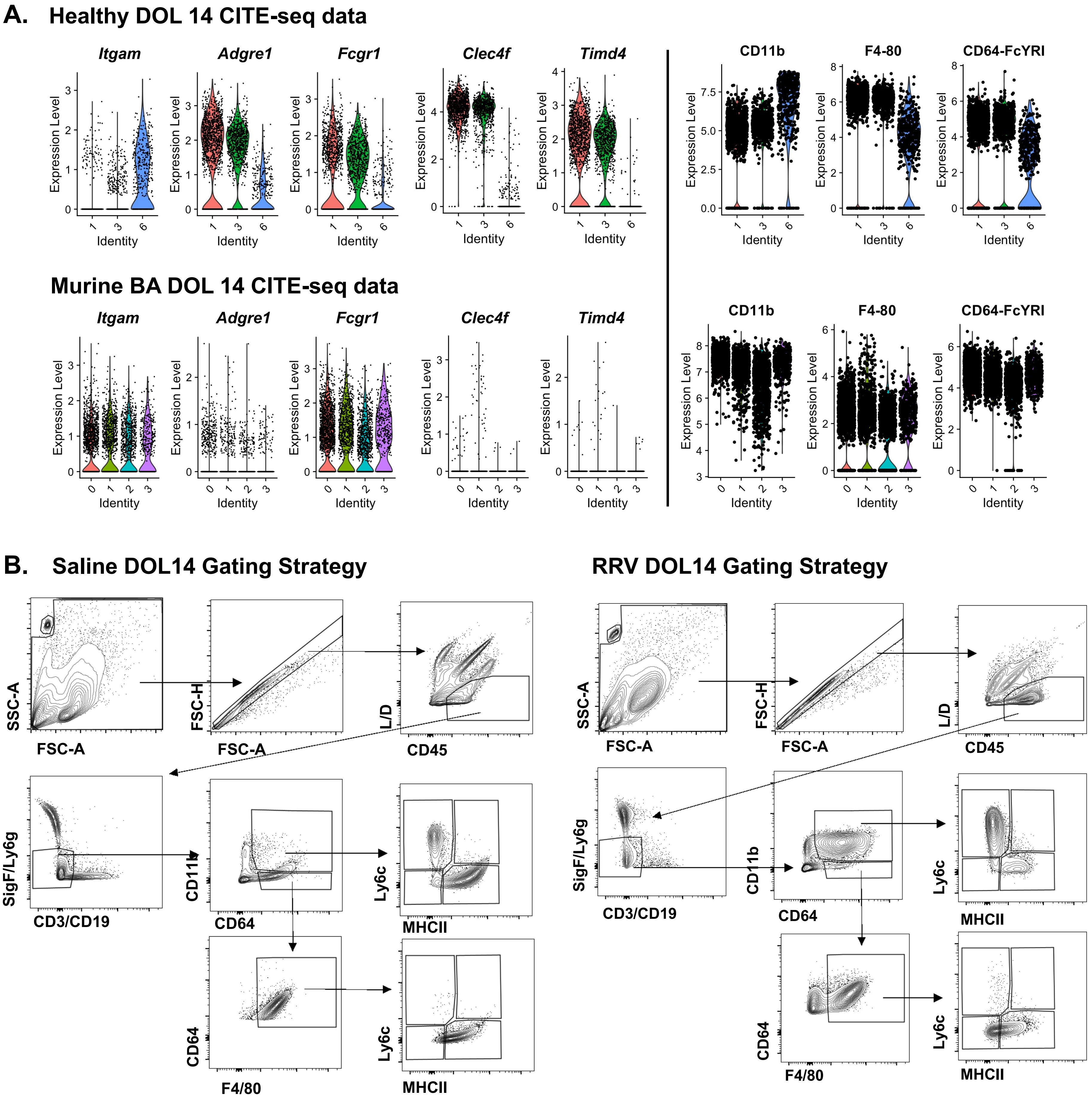

Supplemental Figure 1. Rationale for flow gating strategy for mouse liver. A. Summary of scRNA-seq data (Left) and ADT data (Right) that show CD11b^{lo}Itgam(F4/80)^{hi} tissue resident macrophages (i.e. Kupffer cells) in healthy mice at DOL14 as compared to CD11b^{hi} infiltrating macrophages in murine BA(20). **B.** Gating strategy used for flow cytometry to identify CD11b^{hi}CD64+ macrophages and CD11b^{lo}F4/80+ Kupffer cells in saline controls and murine BA. As expected, CD11b^{lo}F4/80+ Kupffer cells lack Ly6c expression.
DOL – day of life; RRV - Rhesus rotavirus
