# Supplementary material for: Transcriptional analysis of murine biliary atresia identifies macrophage heterogeneity and subset-specific macrophage functions
Source: Front Immunol. 2025 Jan 30;16:1506195. doi: 10.3389/fimmu.2025.1506195 (PMC11821939; doi:10.3389/fimmu.2025.1506195)
Supplement: Supplementary file 9 [file DataSheet2.pdf]

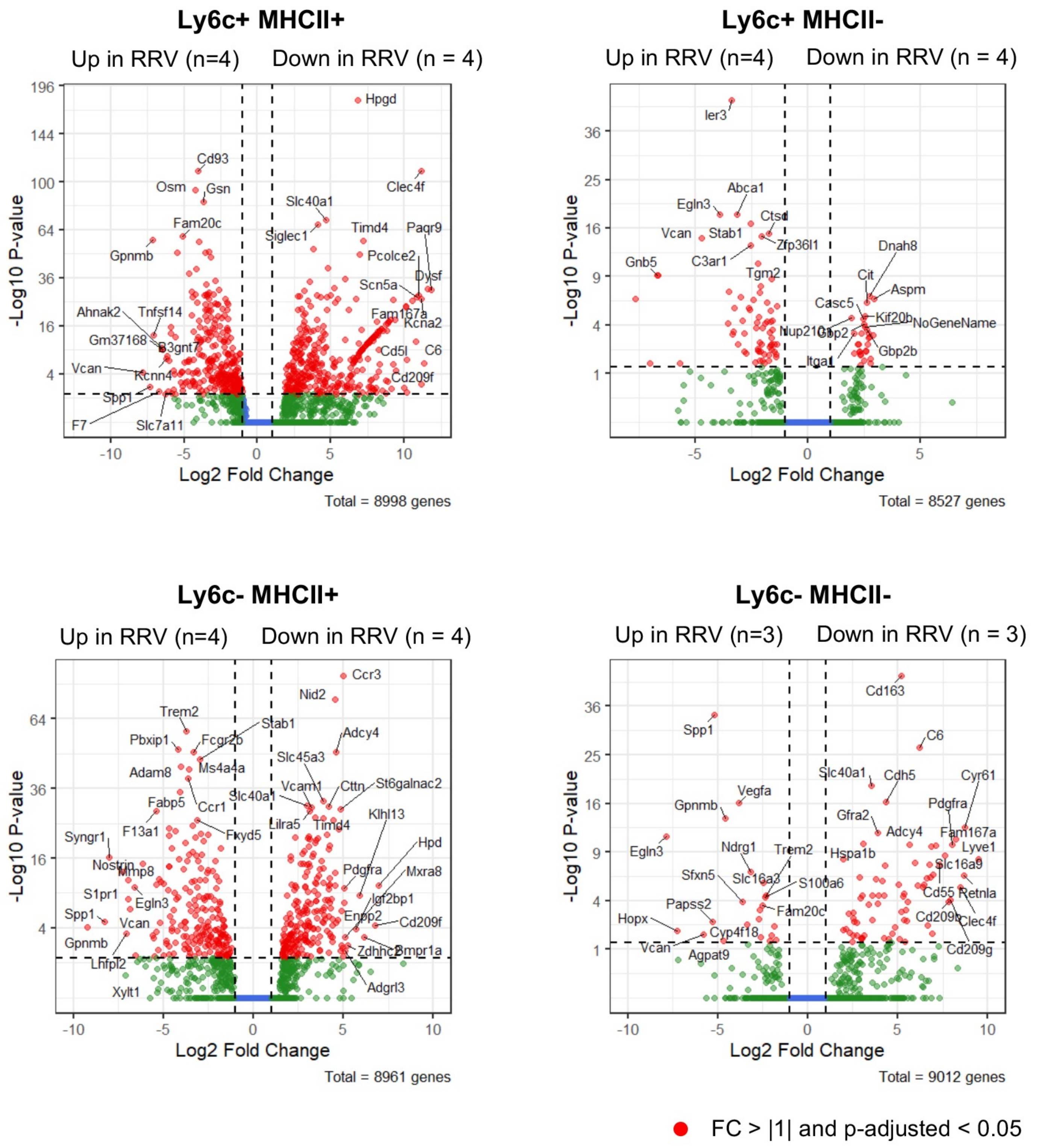

Supplemental Figure 2. Differentially expressed genes for each macrophage subset by experimental condition. Volcano plots show differentially expressed genes (red) in murine BA (RRV) versus saline control by macrophage subset defined by DEseq (p-adjusted <0.05, |log2(FC)|>1). n=4 for all transcriptional comparisons except DN subsets where n=3.
