# Supplementary material for: Transcriptional analysis of murine biliary atresia identifies macrophage heterogeneity and subset-specific macrophage functions
Source: Front Immunol. 2025 Jan 30;16:1506195. doi: 10.3389/fimmu.2025.1506195 (PMC11821939; doi:10.3389/fimmu.2025.1506195)
Supplement: Supplementary file 10 [file DataSheet3.pdf]

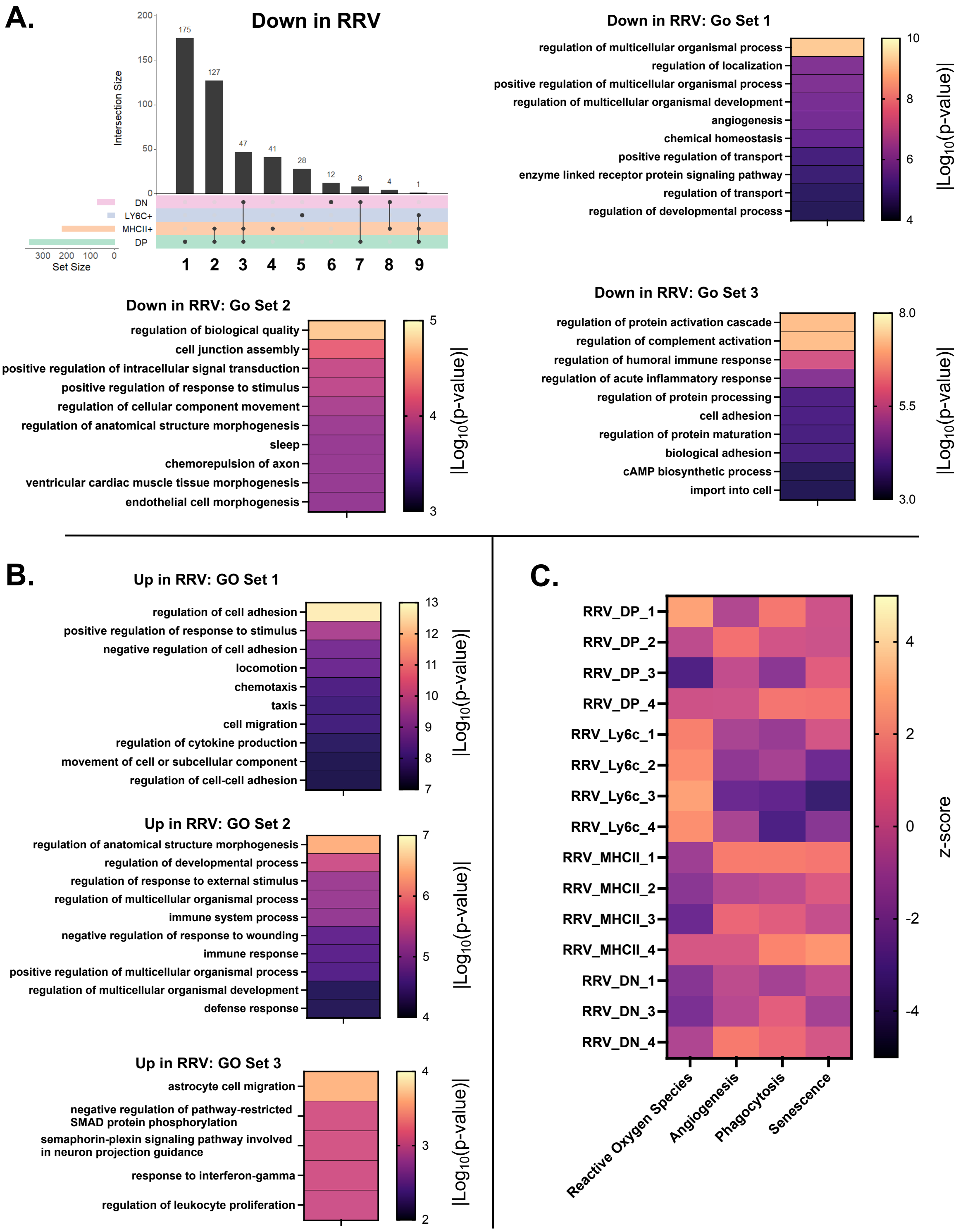

Supplemental Figure 3. Evaluation of gene sets and processes that differ in murine BA. A. The number of differentially expressed genes (DEGs) decreased in murine BA (RRV) versus saline controls and the overlap between subsets (solid line) was calculated using the UpsetR package. Gene ontology enrichment analysis is shown for the first 3 UpSet gene sets decreased in murine BA. **B.** Top 10 processes from gene ontology enrichment analysis of first 3 UpSet gene sets increased in murine BA are shown. **C.** Enrichment for Hallmark Reactive Oxygen Species and Angiogenesis gene sets(27), Mouse Genome Database Phagocytosis engulfment gene set(28-29), and the senescence gene set(30) were evaluated by gene set variation analysis. n=4 for all transcriptional comparisons except DN subsets where n=3.
