# Supplementary material for: Transcriptional analysis of murine biliary atresia identifies macrophage heterogeneity and subset-specific macrophage functions
Source: Front Immunol. 2025 Jan 30;16:1506195. doi: 10.3389/fimmu.2025.1506195 (PMC11821939; doi:10.3389/fimmu.2025.1506195)
Supplement: Supplementary file 11 [file DataSheet4.pdf]

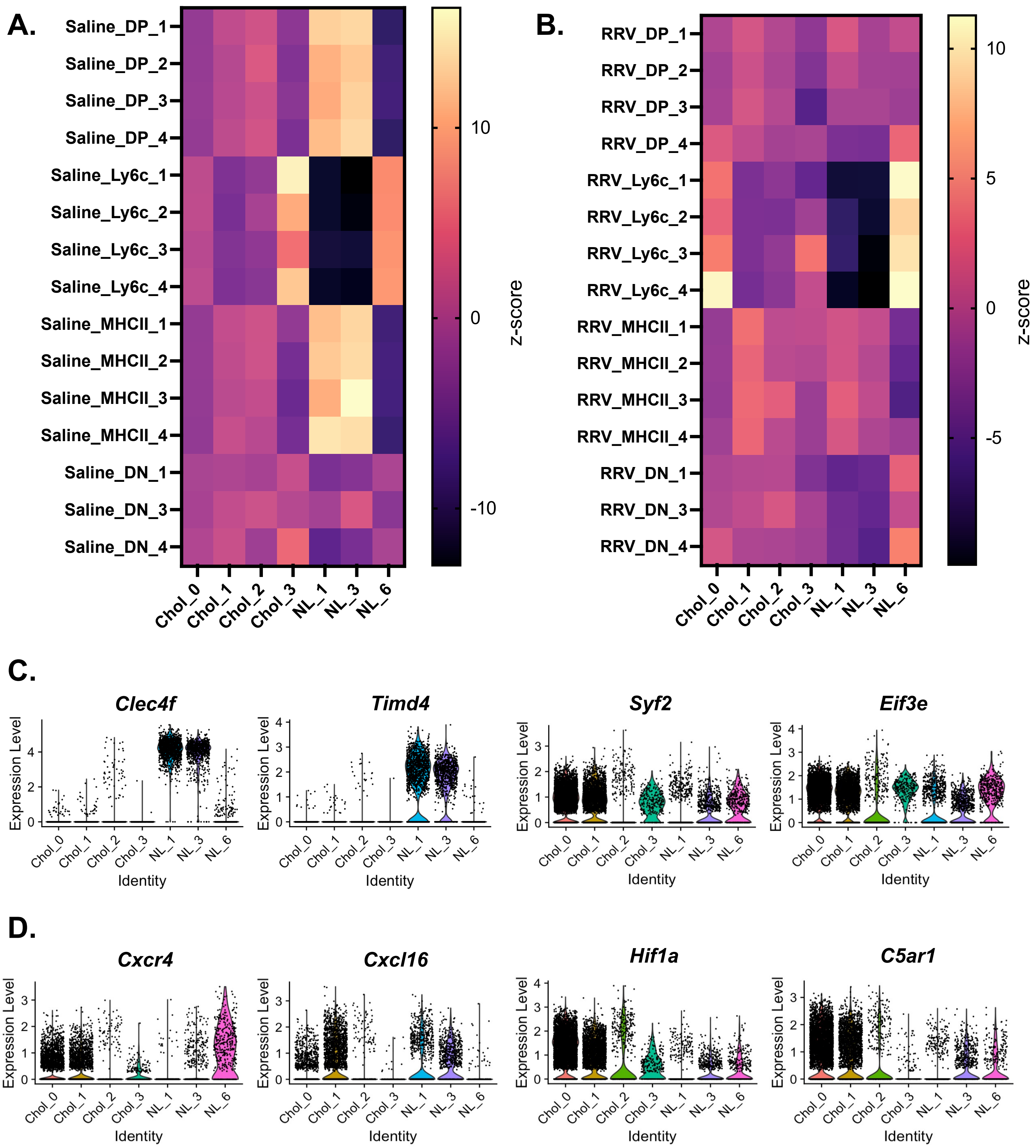

Supplemental Figure 4. Transcriptional comparison between our murine macrophage subsets and prior single-cell sequencing data from cholestatic and normal neonatal mice. A-B. The expression level of differentially expressed genes (DEGs) from our prior single-cell sequencing data(20) was evaluated by gene set variation analysis across our saline control (**A**) and murine BA (**B**) macrophage subsets. **C-D.** Expression of representative genes that differentiated MHCII+ vs Ly6c+ saline (**C**) and murine BA (**D**) macrophages are plotted on prior neonatal murine cholestatic (Chol) and non-diseased (NL) macrophage clusters from single-cell sequencing analysis(20).

Chol – cholestatic macrophage cluster; NL – non-diseased neonatal macrophage cluster
