# Supplementary material for: Transcriptional analysis of murine biliary atresia identifies macrophage heterogeneity and subset-specific macrophage functions
Source: Front Immunol. 2025 Jan 30;16:1506195. doi: 10.3389/fimmu.2025.1506195 (PMC11821939; doi:10.3389/fimmu.2025.1506195)
Supplement: Supplementary file 12 [file DataSheet5.pdf]

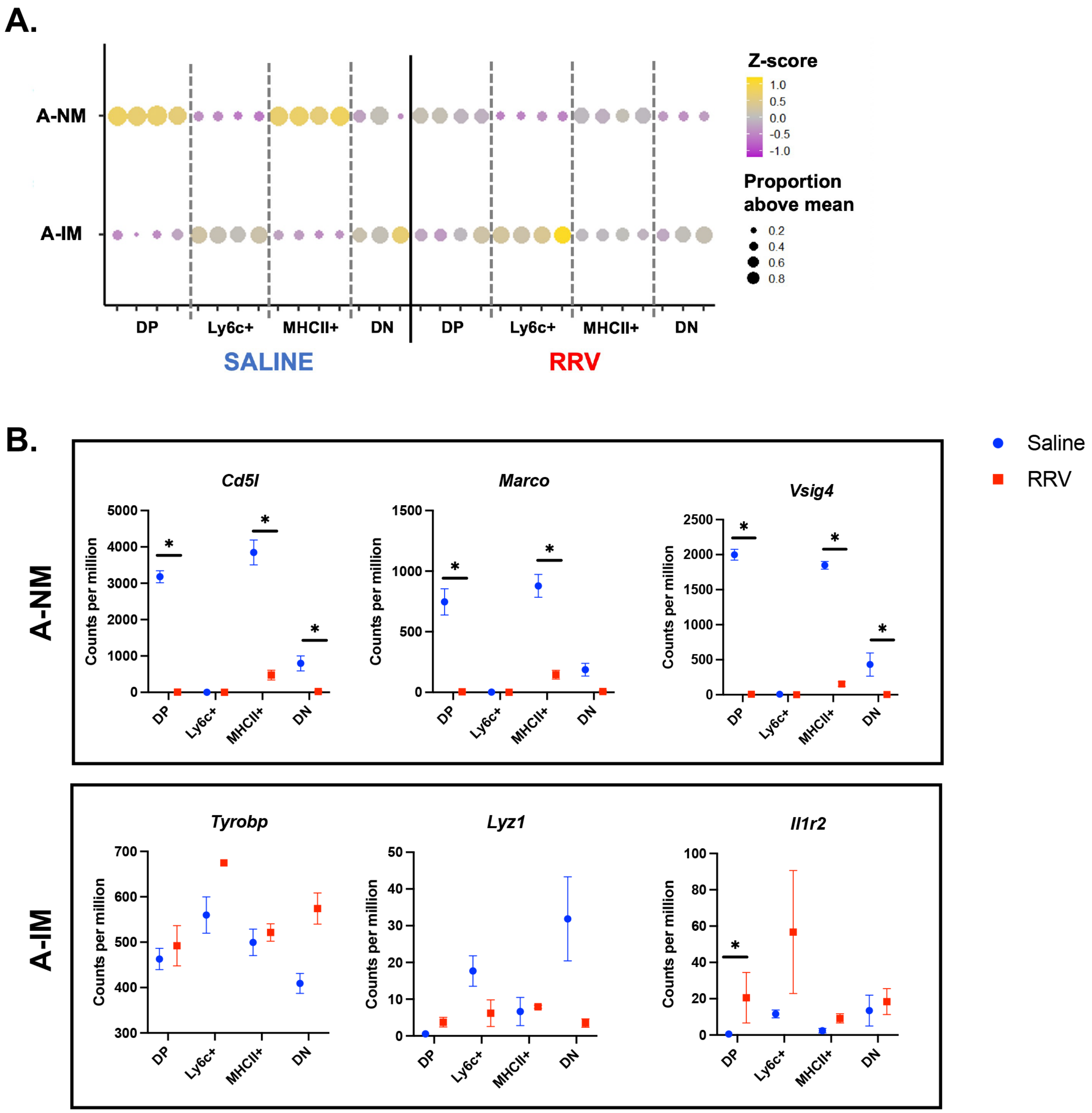

Supplemental Figure 5. Transcriptional comparison between non-diseased human macrophage subsets and our murine macrophages. A. The top 20 genes from adult non-diseased macrophage subsets(13) were converted to their murine orthologs and the mean expression of these genes was visualized across murine BA macrophage subsets. This analysis demonstrated a enrichment for A-NM in saline DP and MHCII+ macrophages were as the A-IM signature was enriched in saline and murine BA Ly6c+ macrophages. **B.** Murine orthologs of representative genes that define A-NM and A-IM subsets are shown across murine macrophage subsets. n=4 for all transcriptional comparisons in murine experiments except DN subsets where n=3. * indicates adjusted p-value < 0.05 by DESeq.

A-IM – adult inflammatory macrophage; A-NM – adult non-inflammatory macrophage; DN – double negative; DP – double positive; RRV - Rhesus rotavirus
