# Supplementary material for: Transcriptional analysis of murine biliary atresia identifies macrophage heterogeneity and subset-specific macrophage functions
Source: Front Immunol. 2025 Jan 30;16:1506195. doi: 10.3389/fimmu.2025.1506195 (PMC11821939; doi:10.3389/fimmu.2025.1506195)
Supplement: Supplementary file 13 [file DataSheet6.pdf]

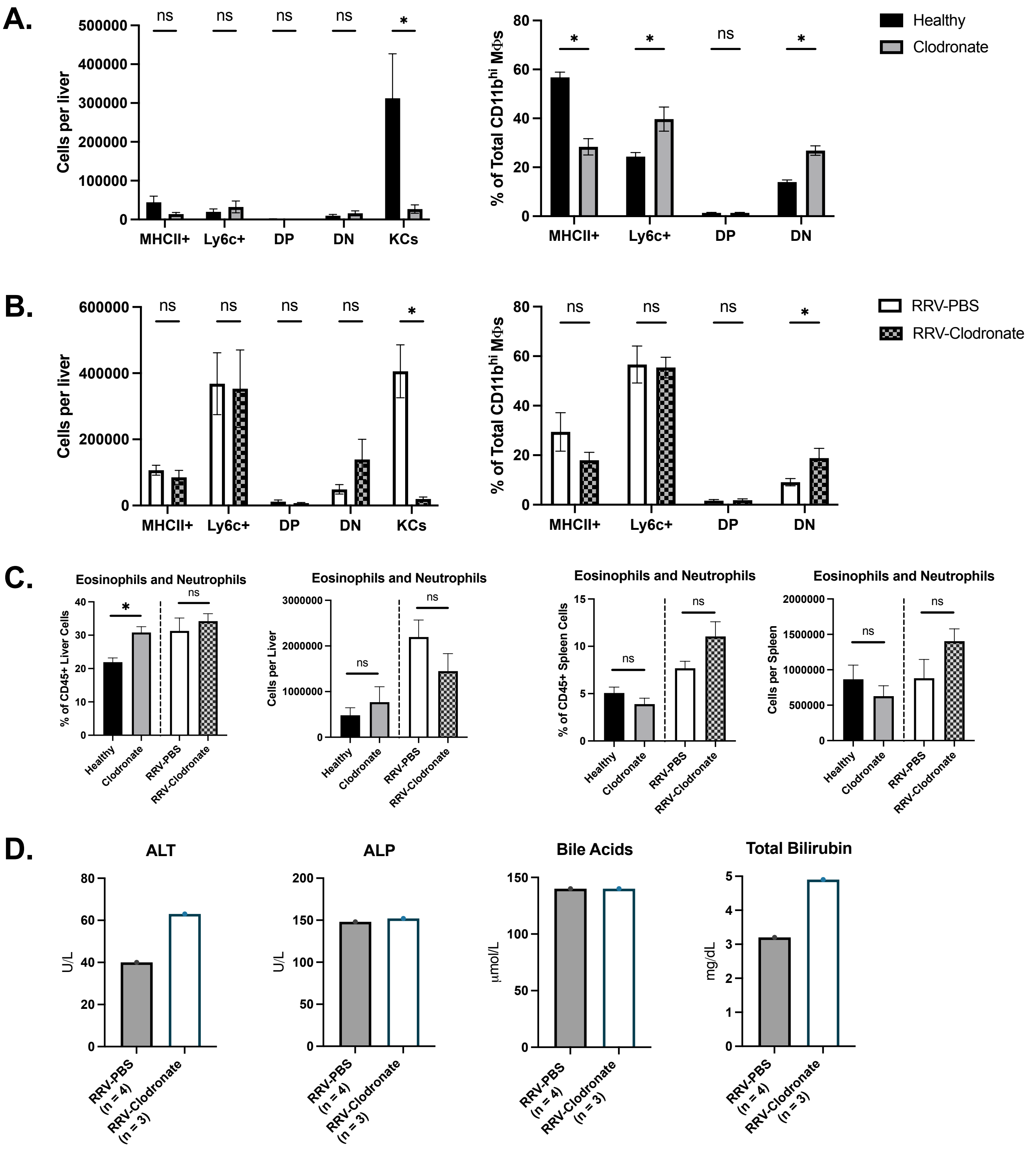

E.

	Total Ishak		Portal Inflammation		Lytic Necrosis		Confluent Necrosis		Piecemeal Necrosis	
	Pearson r	p-value	Pearson r	p-value	Pearson r	p-value	Pearson r	p-value	Pearson r	p-value
**Total CD11bhi MΦ**	0.768	**0.001**	0.735	**0.003**	0.738	**0.003**	0.515	0.060	0.664	**0.010**
**DP**	0.694	**0.006**	0.600	**0.023**	0.716	**0.004**	0.537	**0.048**	0.569	**0.034**
**MHCII+**	0.582	**0.029**	0.503	0.067	0.566	**0.035**	0.386	0.173	0.589	**0.027**
**Ly6c+**	0.835	**<0.001**	0.815	**<0.001**	0.795	**0.001**	0.549	**0.042**	0.718	**0.004**
**DN**	0.247	0.395	0.221	0.447	0.253	0.384	0.182	0.534	0.201	0.491
**KCs**	-0.462	0.096	-0.451	0.105	-0.489	0.076	-0.284	0.325	-0.332	0.246

Supplemental Figure 6. Evaluation of the effect of clodronate-loaded liposomes on macrophage composition in normal and BA mice. A. Clodronate loaded liposomes given to healthy pups resulted in significant reduction in the number of KCs. Among CD11b^{hi} subsets, the proportion of MHCII+ macrophages decreased in clodronate-treated mice compared to healthy controls. **B.** Clodronate treatment in murine BA mice reduced the number of KCs but did not significantly deplete a CD11b^{hi} macrophage subset. **C.** Pooled laboratory data from murine BA mice treated with clodronate or saline showed biochemical evidence of obstructive cholestasis. **D.** Pearson correlation of macrophage numbers and Ishak score (total score and individual components) was performed. All individual CD11b^{hi} subsets aside from DN were directly correlated with total Ishak score. The number of KCs inversely correlated with total Ishak score, however, this did not achieve statistical significance.

* indicates p-value < 0.05 by unpaired Welch's t-test.

ALT – alanine aminotransferase; ALP – alkaline phosphatase; DN – double negative; DP – double positive; PBS - phosphate buffered saline; RRV - Rhesus rotavirus
